# NMR characterization of solvent accessibility and transient structure in intrinsically disordered proteins

**DOI:** 10.1007/s10858-019-00248-2

**Published:** 2019-07-11

**Authors:** Christoph Hartlmüller, Emil Spreitzer, Christoph Göbl, Fabio Falsone, Tobias Madl

**Affiliations:** 1 Center for Integrated Protein Science Munich (CIPSM) at the Department of Chemistry, Technische Universität München, Lichtenbergstrasse 4, 87548 Garching, Germany; 2 Gottfried Schatz Research Center for Cell Signaling, Metabolism and Aging, Institute of Molecular Biology & Biochemistry, Medical University of Graz, Neue Stiftingtalstrasse 6, 8010 Graz, Austria; 3 The Campbell Family Institute for Breast Cancer Research at Princess Margaret Cancer Centre, 610 University Avenue, Toronto, ON M5G 2M9 Canada; 4 Institute of Pharmaceutical Sciences, University of Graz, Schubertstrasse 1, 8010 Graz, Austria; 5 BioTechMed-Graz, Graz, Austria

**Keywords:** Solvent paramagnetic relaxation enhancement, Intrinsically disordered proteins, Residual structure, p53, FOXO4, α-Synuclein

## Abstract

In order to understand the conformational behavior of intrinsically disordered proteins (IDPs) and their biological interaction networks, the detection of residual structure and long-range interactions is required. However, the large number of degrees of conformational freedom of disordered proteins require the integration of extensive sets of experimental data, which are difficult to obtain. Here, we provide a straightforward approach for the detection of residual structure and long-range interactions in IDPs under near-native conditions using solvent paramagnetic relaxation enhancement (sPRE). Our data indicate that for the general case of an unfolded chain, with a local flexibility described by the overwhelming majority of available combinations, sPREs of non-exchangeable protons can be accurately predicted through an ensemble-based fragment approach. We show for the disordered protein α-synuclein and disordered regions of the proteins FOXO4 and p53 that deviation from random coil behavior can be interpreted in terms of intrinsic propensity to populate local structure in interaction sites of these proteins and to adopt transient long-range structure. The presented modification-free approach promises to be applicable to study conformational dynamics of IDPs and other dynamic biomolecules in an integrative approach.

**Electronic supplementary material:**

The online version of this article (10.1007/s10858-019-00248-2) contains supplementary material, which is available to authorized users.

## Introduction

The well-established structure–function paradigm has been challenged by the discovery of intrinsically disordered proteins (IDPs) (Dyson and Wright 2005). It is suggested that about 40% of all proteins have disordered regions of 40 or more residues, with many proteins existing solely in the unfolded state (Tompa 2012; Romero et al. 1998). Although they lack stable secondary or tertiary structure elements, this large class of proteins plays a crucial role in various cellular processes (Theillet et al. 2014; Wright and Dyson 2015; van der Lee et al. 2014; Uversky et al. 2014). Disorder serves a biological role, where conformational heterogeneity granted by disordered regions enables proteins to exert diverse functions in response to various stimuli. Unlike structured proteins, which are essential for catalysis and transport, disordered proteins are crucial for regulation and signaling. Due to their intrinsic flexibility they can act as network hubs interacting with a wide range of biomolecules forming dynamic regulatory networks (Dyson and Wright 2005; Tompa 2012; Babu et al. 2011; Flock et al. 2014; Wright and Dyson 1999; Uversky 2011; Habchi et al. 2014). Given the plethora of potential interaction partners, it is not surprising that the interaction of IDPs with binding partners are often tightly regulated via and intricate ‘code’ of post-translational modifications, including phosphorylation, methylation, acetylation, and various others (Wright and Dyson 2015; Bah and Forman-Kay 2016). These proteins, and distortions in their interaction networks, for example by mutations and aberrant post-translational modifications (PTMs), are closely linked to a range of human diseases, including cancers, neurodegeneration, cardiovascular disorders and diabetes, they are currently considered difficult to study (Dyson and Wright 2005; Tompa 2012; Babu et al. 2011; Habchi et al. 2014; Metallo 2010; Uversky et al. 2008; Dyson and Wright 2004). Complications arise from the following factors: these proteins lack well-defined stable structure, they exist in a dynamic equilibrium of distinct conformational states, and the number of experimental techniques and observables renders IDP conformational characterization underdetermined (Mittag and Forman-Kay 2007; Eliezer 2009). Thus, an integration of new sets of experimental and analytical techniques are required to characterize the conformational behavior of IDPs.

Although IDPs are highly dynamic, they often contain transiently-folded regions, such as transiently populated secondary or tertiary structure, transient long-range interactions or transient aggregation (Marsh et al. 2007; Shortle and Ackerman 2001; Bernado et al. 2005; Mukrasch et al. 2007; Wells et al. 2008). These transiently-structured regions are of particular interest to study the biological function of IDPs as they can report on biologically-relevant interactions and encode biological function. Examples are aggregation, liquid–liquid phase separation, binding to folded co-factors, or modifying enzymes (Yuwen et al. 2018; Brady et al. 2017; Choy et al. 2012; Maji et al. 2009; Putker et al. 2013).

NMR spectroscopy is exceptionally well-suited to study IDPs, and in particular to detect transiently folded regions (Meier et al. 2008; Wright and Dyson 2009; Jensen et al. 2009). Several NMR observables provide atomic resolution, and ensemble-averaged information reporting on the conformational energy landscape sampled by each amino acid, including chemical shifts, residual dipolar couplings (RDCs), and paramagnetic relaxation enhancement (PRE) (Dyson and Wright 2004; Eliezer 2009; Marsh et al. 2007; Shortle and Ackerman 2001; Meier et al. 2008; Gobl et al. 2014; Gillespie and Shortle 1997; Clore et al. 2007; Huang et al. 2014; Ozenne et al. 2012; Clore and Iwahara 2009; Otting 2010; Hass and Ubbink 2014; Gobl et al. 2016). RDCs, and PREs, either alone or in combination have been used successfully in recent years to characterize the conformations and long-range interactions of IDPs (Bernado et al. 2005; Ozenne et al. 2012; Dedmon et al. 2005; Bertoncini et al. 2005; Parigi et al. 2014; Rezaei-Ghaleh et al. 2018). However, both techniques rely on a modification of the IDP of interest, either by external alignment media in case of RDCs or the covalent incorporation of paramagnetic tags in the case of PREs.

**Fig. 1:**
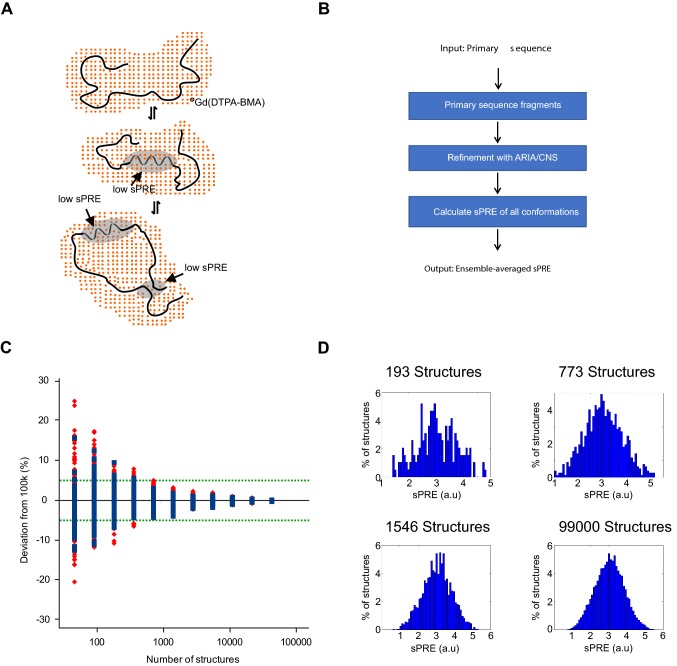
Principle and workflow for solvent PRE. **a** Transient secondary structures of IDPs are characteristic for protein–protein interaction sites and are therefore crucial for various cellular functions. NMR sPRE data provide quantitative and residue specific information on the solvent accessibility as the effect of paramagnetic probes such as Gd(DTPA-BMA) is distance dependent, which can be used to detect secondary structures within otherwise unfolded regions and long-range contacts within a protein. **b** Prediction of sPRE is based on an ensemble approach of a library of peptides. Each peptide has a length of 5 residues, and is flanked by triple-Ala on both termini (e.g. AAAXXXXXAAA, where XXXXX is a 5-mer fragment of the target primary sequence). Following water refinement using ARIA/CNS, sPRE values of all conformations are calculated and the average solvent PRE value of the ensemble is returned. **c** Predicted C^α^ sPRE (blue) and standard deviation (red) of AAAVVAVVAAA ensembles consisting of 99,000 down to 48 structural conformations. The green-dotted line indicates 5% deviation from the ensemble with 99,000 conformations. **d** Histograms of different ensemble sizes showing the distribution of predicted sPRE values

We and others have proposed applications of soluble paramagnetic agents to obtain structural information by NMR without any modifications of the molecules of interest (Gobl et al. 2014; Guttler et al. 2010; Hartlmuller et al. 2016; Hocking et al. 2013; Madl et al. 2009, 2011; Respondek et al. 2007; Zangger et al. 2009; Pintacuda and Otting 2002; Bernini et al. 2009; Wang et al. 2012; Sun et al. 2011; Gong et al. 2017; Gu et al. 2014; Hartlmuller et al. 2017). The addition of soluble paramagnetic compounds leads to a concentration-dependent and therefore tunable increase of relaxation rates, the so-called paramagnetic relaxation enhancement (here denoted as solvent PRE, sPRE; also known as co-solute PRE, Fig. 1a). This effect depends on the distance of the spins of interest (e.g. ^1^H, ^13^C) to the biomolecular surface. The nuclei on the surface are affected the strongest by the sPRE effect, and this approach has been shown to correlate well with biomolecular structure in the case of proteins and RNA (Madl et al. 2009; Pintacuda and Otting 2002; Bernini et al. 2009; Hartlmuller et al. 2017). sPREs have gained popularity for structural studies of biomolecules, including in the structure determination of proteins (Madl et al. 2009; Wang et al. 2012), docking of protein complexes (Madl et al. 2011), and qualitative detection of dynamics (Hocking et al. 2013; Sun et al. 2011; Gong et al. 2017; Gu et al. 2014).

The most commonly used paramagnetic agent for measuring sPRE data is the inert complex Gd(DTPA-BMA) (gadolinium diethylenetriaminepenta-acetic acid bismethylamide, commercially available as ‘Omniscan’), that is known to not specifically interact on protein surfaces (Guttler et al. 2010; Madl et al. 2009, 2011; Pintacuda and Otting 2002; Wang et al. 2012; Respondek et al. 2007; Zangger et al. 2009; Göbl et al. 2010). Previously, we and others could show that sPRE data provide in-depth structural and dynamic data for IDP analysis (Madl et al. 2009; Sun et al. 2011; Gong et al. 2017; Emmanouilidis et al. 2017; Johansson et al. 2014). For example, sPRE data helped to characterize α-helical propensity in a previously postulated flexible region in the folded 42 kDa maltodextrin binding protein (Madl et al. 2009), and dynamic ligand binding to the human “survival of motor neuron” protein (Emmanouilidis et al. 2017). While writing this manuscript, and based on sPRE data for exchangeable amide protons, the Tjandra lab has shown that sPREs can detect native-like structure in denatured ubiquitin (Kooshapur et al. 2018).

Here, we present an integrative ensemble approach to predict the sPREs of IDPs. This ensemble representation is used to calculate conformationally averaged sPREs, which fit remarkably well to the experimentally-measured sPREs. We show for the disordered protein α-synuclein, and disordered regions of the proteins FOXO4 and p53 that deviation from random coil behavior can indicate intrinsic propensity to populate transient local structures and long-range interactions. In summary, this method provides a unique modification-free approach for studying IDPs, that is compatible with a wide range of NMR pulse sequences and biomolecules.

## Materials and methods

### Protein expression and purification

For expression of human FOXO4^TAD^ (residues 198–505), p53^TAD^ (residues 1–94), pETM11-His_6_-ZZ cDNA and including an N-terminal TEV protease cleavage site coding for the respective proteins were transformed into *E. coli* BL21-DE3. To obtain ^13^C/^15^N isotope labeled protein, cells were grown for 1 day at 37 °C in minimal medium (100 mM KH_2_PO_4,_ 50 mM K_2_HPO_4,_ 60 mM Na_2_HPO_4,_ 14 mM K_2_SO_4,_ 5 mM MgCl_2_; pH 7.2 adjusted with HCl and NaOH with 0.1 dilution of trace element solution (41 mM CaCl_2,_ 22 mM FeSO_4,_ 6 mM MnCl_2,_ 3 mM CoCl_1,_ 2 mM ZnSO_4_, 0.1 mM CuCl_2_, 0.2 mM (NH_4_)_6_Mo_7_O_17,_ 24 mM EDTA) supplemented with 2 g of ^13^C_6_H_12_O_6_ (Cambridge isotope) and 1 g of ^15^NH_4_Cl (Sigma). At an OD (600 nm) of 0.8, cells were induced with 0.5 mM isopropyl-β-d-thiogalactopyranosid (IPTG) for 16 h at 20 °C. Cell pellets were harvested and sonicated in denaturing buffer containing 50 mM Tris–HCl pH 7.5, 150 mM NaCl, 20 mM imidazole, 2 mM tris(2-carboxyethyl)phosphine (TCEP), 20% glycerol and 6 M urea. His_6_-ZZ proteins were purified using Ni–NTA agarose (QIAGEN) and eluted in 50 mM Tris–HCl pH 7.5, 150 mM NaCl, 200 mM imidazole, 2 mM TCEP and subjected to TEV protease cleavage. Untagged proteins were then isolated by performing a second affinity purification step using Ni–NTA beads for removal of TEV and uncleaved substrate. A final exclusion chromatography purification step was performed in the buffer of interest on a gel filtration column (Superdex peptide (10/300) for p53 and Superdex 75 (16/600) for FOXO4, GE Healthcare).

α-Synuclein was expressed and purified as described (Falsone et al. 2011). Briefly, pRSETB vector containing the human AS gene was transformed into BL21 (DE3) Star Cells. ^13^C/^15^N-labeled α-synuclein was expressed in minimal medium (6.8 g/l Na_2_HPO_3,_ 4 g/l KH_2_PO_4_, 0.5 g/l NaCl, 1.5 g/l (15NH_4_)_2_SO_2,_ 4 g/l ^13^C glucose, 1 μg/l biotin, 1 μg/l thiamin, 100 μg/ml ampicillin, and 1 ml 1000 × microsalts). Cells were grown to an OD (600 nm) of 0.7. Protein was expressed by addition of 1 mM IPTG for 4 h. After harvesting cells were resuspended in 20 mM Tris–HCl, 50 mM NaCl, pH 7.4, supplemented with a Complete^®^ protease inhibitor mix (Roche, Basel, Switzerland). Protein purification was then achieved using a Resource Q column and gel filtration using a Superdex 75 gel filtration column (GE Healthcare, Uppsala, Sweden).

### Generation of conformational ensembles

Conformational ensembles were generated using the ARIA/CNS software-package, comprising of 1500 random backbone conformations of all possible 5-mer peptides of the protein of interest, and flanked by triple-alanine. Every backbone conformation served as starting structure in a full-atom water refinement using ARIA (Bardiaux et al. 2012). For every refined structure the solvent PRE is calculated and the averaged solvent PRE of the central residue is stored in the database. To predict sPRE data, a previously published grid-based approach was used (Hartlmuller et al. 2016; Pintacuda and Otting 2002). Briefly, the structural model was placed in a regularly-spaced grid representing the uniformly distributed paramagnetic compound and the grid was built with a point-to-point distance of 0.5 Å and a minimum distance of 10 Å between the protein model and the outer border of the grid. Next, grid points that overlap with the protein model were removed assuming a molecular radius of 3.5 Å for the paramagnetic compound. To compute the sPRE for a given protein proton ${\text{sPRE}}_{\text{predicted}}^{i}$, the distance-dependent paramagnetic effect (Hartlmuller et al. 2016; Hocking et al. 2013; Pintacuda and Otting 2002) was numerically integrated over all remaining grid points according to Eq. (1):

1 $${\text{sPRE}}_{\text{predicted}}^{i} = c \cdot \mathop \sum \limits_{{d_{i,j} < 10 \AA}} \frac{1}{{d_{i,j}^{6} }}$$

where *i* is the index of a proton of the protein, *j* is the index of the grid point, *d*_*i, j*_ is the distance between the ith proton and the jth grid point and *c* is an arbitrary constant to scale the sPRE values (1000). Theoretical sPRE values were normalized by calculating the linear fit of experimental and predicted sPRE followed by shifting and scaling of the theoretical sPRE. To predict the solvent PRE of the entire IDP sequence, each peptide with the five matching amino acids is searched and the corresponding solvent PRE values are combined. sPRE data of the two N- and C-terminal residues were not predicted in this setup. All scripts and sample runs can be obtained downloaded from the homepage of the authors (https://mbbc.medunigraz.at/forschung/forschungseinheiten-und-gruppen/forschungsgruppe-tobias-madl/software/).

### NMR experiments

The setup of sPRE measurements using NMR spectroscopy was performed as described previously (Hartlmuller et al. 2016, 2017). To obtain sPRE data, a saturation-based approach was used. The ^1^H-R_1_ relaxation rates were determined by a saturation-recovery scheme followed by a read-out experiment such as a ^1^H, ^15^N HSQC, ^1^H, ^13^C HSQC or a 3D CBCA(CO)NH experiment. The read-out experiments were combined with the saturation-recovery scheme in a Pseudo-3D (HSQCs) or Pseudo-4D [CBCA(CO)NH] experiment, with the recovery time as an additional dimension. The CBCA(CO)NH was recorded using non-uniform sampling. Alternatively, ^1^H-R_2_ relaxation rates can be as described (Clore and Iwahara 2009).

A 7.5 ms ^1^H trim pulse followed by a gradient was applied for proton saturation. During the recovery, ranging from several milliseconds up to several seconds, z-magnetization is built up. The individual recovery delays are applied in an interleaved manner, with short and long delays occurring in alternating fashion. For every ^1^H-R_1_ measurement 10 delay times were recorded and for error estimation, at 1 delay time was recorded as a duplicate.

Measurement of ^1^H-R_1_ rates were repeated for increasing concentrations of the relaxation-enhancing Gd(DTPA-BMA)/Omniscan (GE Healthcare, Vienna, Austria) and the solvent PRE was obtained as the average change of the proton R_1_ rate per concentration of the paramagnetic agent. After each addition of Gd(DTPA-BMA), the recovery delays were shortened such that for the longest delay all NMR signals were sufficiently recovered. The interscan delay was set to 50 ms, as the saturation-recovery scheme does not rely on an equilibrium z-magnetization at the start of each scan. All NMR samples contained 10% ^2^H_2_O. Spectra were processed using NMRPipe and analyzed with the NMRView and CcpNmr Analysis software packages (Johnson 2004; Delaglio et al. 1995; Skinner et al. 2016).

### Measurement of sPRE data used in this study

Assignment of p53^TAD^ was achieved using HNCACB, CBCA(CO)NH and HCAN spectra and analyzed using ccpNMR (Skinner et al. 2016). sPRE data of 300 µM samples of uniformly ^13^C/^15^N labeled p53^TAD^ was measured on a 600 MHz Bruker Avance Neo NMR spectrometer equipped with a TXI probehead at 298 K in a buffer containing 50 mM sodium phosphate buffer, 0.04% sodium azide, pH 7.5. ^1^H-R_1_ rates of ^1^H^N^, H^α^ and H^β^ were determined using ^1^H,^13^C HSQC and ^1^H, ^15^N HSQC as read-out spectra (4/4 scans, 200/128 complex points in F2). For assignment of α-synuclein, previously reported chemical shifts were obtained from the BMRB (accession code 6968) and the assignment was confirmed using HNCACB and CBCA(CO)NH spectra. ^1^H-R_1_ rates of aliphatic protons and amide protons of a 100 µM sample (50 mM bis(2-hydroxyethyl)amino-tris(hydroxymethyl)methane (bis–Tris), 20 mM NaCl, 3 mM sodium azide, pH 6.8) were determined using ^1^H, ^13^C HSQC and ^1^H, ^15^N HSQC read-out spectra, respectively, at 282 K in the presence of 0, 1, 2, 3, 4 and 5 mM Gd(DTPA-BMA). For assignment of FOXO4^TAD^ HNCACB, CBCA(CO)NH and HCAN spectra were recorded and assigned using ccpNMR (Skinner et al. 2016). Measurements of ^13^C, ^15^N labeled FOXO4^TAD^ at 390 µM in 20 mM sodium phosphate buffer, pH 6.8, 50 mM NaCl, 1 mM DTT were performed on a 600 MHz magnet (Oxford Instruments) equipped with an AV III console and cryo TCI probe head (Bruker Biospin). Pseudo-4D CBCA(CO)NH spectra served as read-out for ^1^H-R_1_ rates and were recorded on a 250 µM sample on a 900 MHz Bruker Avance III spectrometer equipped with a TCI cryoprobe using non-uniform sampling (4 scans, 168/104 complex points in F1 (^13^C)/F2 (^15^N) sampled with 13.7% resulting a total number of 600 complex points). Spectra were processed using hmsIST/NMRPipe (Hyberts et al. 2014).

### Analysis of NMR data

sPRE data of the model proteins was analyzed as described previously. Briefly, peak intensities were extracted using nmrglue python package and fitted to a mono-exponential build up curve the SciPy python package and Eq. (2).

2 $$I\left( t \right) = - A \cdot e^{{ - R_{1} *t}} + C$$

where *I*(*t*) is the peak intensity of the saturation-recovery experiment, *t* is the recovery delay, *A* is the amplitude of the z-magnetization build-up, C is the plateau of the curve and *R*_*1*_ is the longitudinal relaxation rate. Duplicate recovery delays were used to determine the error for the fitted rates *R*_*1*_.

3 $$\varepsilon_{ \exp } = \sqrt {\frac{1}{2N} \cdot \mathop \sum \limits_{i = 1}^{N} \delta_{i} }$$

where *N* is the number of peaks in the spectrum, *i* is the index of the peak, and δ_i_ is the difference of the duplicates for the *i*th peak. The error of the rates *R*_1_ was then obtained using a Monte Carlo-type resampling strategy. The solvent PRE is obtained by performing a weighted linear regression using the equation

4 $$R_{1} \left( c \right) = sPRE \cdot c + R_{1}^{0}$$

where *c* is the concentration of Gd(DTPA-BMA), *R*_1_(*c*) is the fitted *R*_1_ rate at the present of Gd(DTPA-BMA) with a concentration *c*, *R*_1_^0^ is the *R*_1_ in the absence of Gd(DTPA-BMA) and sPRE is the slope and the desired sPRE value. For the weighted linear regression, the previously determined errors ∆ *R*_1_ for *R*_1_ was used, and the error on the concentration *c* was neglected.

## Results and discussion

To detect transient structural elements in IDPs, an efficient back-calculation of sPREs of IDPs is essential. Whereas back-calculation of sPREs is relatively straightforward for folded rigid structures and can be carried out efficiently using a grid-based approach by integration of the solvent environment (Hartlmuller et al. 2016, 2017), this approach fails in the case of highly conformationally heterogeneous IDPs. In our approach, sPREs are best represented as an average sPRE of an ensemble. NMR observables and nuclear spin relaxation phenomena, including sPREs, directly sense chemical exchange through the distinct magnetic environments that nuclear spins experience while undergoing those exchange processes. The effects of the dynamic exchange on the NMR signals can be described by the McConnell Equations (Mcconnell 1958) In the case of a two-site exchange process, and assuming that the exchange rate is faster than the difference in the sPREs observed in both states, the observed sPRE is a linear, population-weighted average of the sPRE observed in both states, as seen for covalent paramagnetic labels (Clore and Iwahara 2009). Moreover, the correlation time for relaxation is assumed to be faster than the exchange time among different conformations within the IDP (Jensen et al. 2014; Iwahara and Clore 2010). The effective correlation time for longitudinal relaxation depends on the rotational correlation time of the biomolecule, the electron relaxation time and the lifetime of the rotationally correlated complex of the biomolecule and the paramagnetic agent (Madl et al. 2009; Eletsky et al. 2003). For ubiquitin, the effective correlation time for longitudinal relaxation was found to be in the sub-ns time scale (Pintacuda and Otting 2002), whereas that conformational exchange in IDPs typically appears at slower timescales (Jensen et al. 2014).

Calculating the average of sPREs over an ensemble of protein conformations presents serious practical difficulties that affect both the accuracy and the portability of the calculation. For RDCs it has been shown that convergence to the average requires an unmanageably large number of structures (e.g. 100,000 models for a protein with 100 amino acids), and that the convergence strictly depends on the length of the protein (Bernado et al. 2005; Nodet et al. 2009). To simplify the back-calculation of sPREs we use a strategy proposed for RDCs by the Forman-Kay and Blackledge groups (Marsh et al. 2008; Huang et al. 2013).

To back-calculate the sPRE from a given primary sequence of an IDP we generated fragments of five amino acids of the sequence of interest and flanked them with triple-alanine sequences at the N- and C-termini to simulate the presence of upstream/downstream amino acids (Fig. 1b). An ensemble of structures for these sequences is then generated using ARIA/CNS including water refinement (Bardiaux et al. 2012). To predict the solvent PRE of the entire IDP, the peptide with the five matching residues is searched and the corresponding solvent PREs averaged for the entire conformational ensemble are returned. This approach is highly parallelizable and dramatically reduces the computational effort compared to simulating the conformations of the full-length IDP.

To determine the number of conformers necessary to converge the back-calculated sPRE of the defined 11-mers, we generated an ensemble of 100,000 structures for a 11-mer AAAVVAVVAAA peptide using ARIA/CNS (Bardiaux et al. 2012) and back-calculated the sPRE for subsets with decreasing number of structures. We find that 1500 conformers are sufficient to reproduce the sPRE with a deviation compared to the maximum ensemble below 5% (Fig. 1c, d).

Back-calculation of the sPRE by fast grid-based integration has some advantages compared to alternative approaches relying on surface accessibility (Kooshapur et al. 2018). First, sPREs can be obtained for atoms without any surface accessibility in grid-based integration approaches as they still take into account the distance-dependent paramagnetic effect. This is expected to provide more accurate predictions for regions with a high degree of bulky side chains or transient folding.

To validate our computational approach, we recorded several sets of experimental ^1^H-sPREs for the disordered regions of the human proteins FOXO4, p53, and α-synuclein. Similar to many other transcription factors, p53 and FOXO4 are largely disordered outside their DNA binding domains.

In order to demonstrate that surface accessibility data can be obtained for a challenging IDP, we recorded sPRE data for the 307 residue transactivation domain of FOXO4. The FOXO4 transcription factor is a member of the forkhead box O family of proteins that share a highly conserved DNA-binding motif, the forkhead box domain (FH). The FH domain is surrounded by large N- and C-terminal intrinsically disordered regions which are essential for the regulation of FOXO function (Weigel and Jackle 1990). FOXOs control a plethora of cellular functions, such as cell growth, survival, metabolism and oxidative stress, by regulating the expression of hundreds of target genes (Burgering and Medema 2003; Hornsveld et al. 2018). Expression and activity of FOXOs are tightly controlled by PTMs such as phosphorylation, acetylation, methylation and ubiquitination, and these modifications impact on FOXO stability, sub-cellular localization and transcriptional activity (Essers et al. 2004; de Keizer et al. 2010; van den Berg et al. 2013). Because of their anti-proliferative and pro-apoptotic functions, FOXOs have been considered as bona fide tumor suppressors. However, FOXOs can also support tumor development and progression by maintaining cellular homeostasis, facilitating metastasis and inducing therapeutic resistance (Hornsveld et al. 2018). Thus, targeting FOXO activity might hold promise in cancer therapy.

**Fig. 2:**
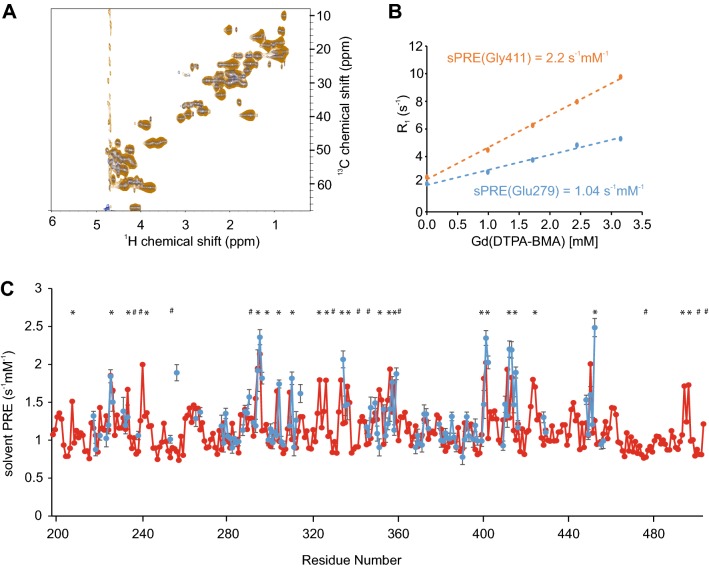
Comparison of predicted and measured solvent PRE of FOXO4^TAD^. **a** Overlay of ^1^H,^13^C HSQC spectra, with full recovery time of a 390 µM ^13^C,^15^N labeled FOXO4^TAD^ sample in the absence (blue) and presence of 3.25 mM Gd(DTPA-BMA) (orange). **b**^1^H-R_1_ rates of two selected residues of FOXO4^TAD^ at different Gd(DTPA-BMA) concentrations. **c** Predicted (red) and experimentally-determined (blue) solvent PRE values using CBCA(CO)NH as readout spectrum, of assigned H^α^ peaks of FOXO4^TAD^. Experimental sPRE values are calculated by fitting the data with a linear regression equation. Predicted sPRE values are based on the previously described ensemble approach. Residues with bulky side chains (Phe, Trp, Tyr) are labeled with #, and exposed glycine residues are labeled with * (see Supporting Fig. 2A for a bulkiness profile). Errors of the measured ^1^H-R_1_ rates were calculated using a Monte Carlo-type resampling strategy and are shown in the diagram as error bars

The C-terminal FOXO4 transactivation domain has been suggested to be largely disordered and to be the binding site for many cofactors. Because it also harbors most of the post-translational modifications (Putker et al. 2013; Burgering and Medema 2003; Hornsveld et al. 2018; Bourgeois and Madl 2018), we set off to study this biologically important domain using our sPRE approach. ^1^H,^15^N and ^1^H, ^13^C HSQC NMR spectra of FOXO4^TAD^ are of high quality and showed no detectable ^1^H, ^13^C, or ^15^N chemical shift changes between the spectra recorded in the absence or presence of Gd(DTPA-BMA) (Fig. 2a). sPRE data of FOXO4 had to be recorded in pseudo-4D saturation-recovery CBCA(CO)NH spectra due to the severe signal overlap observed in the 2D HSQC spectra. It should be noted that any kind of NMR experiment could be combined in principle with a sPRE saturation recovery measurement block in order to obtain ^1^H- or ^13^C sPRE data. The sPRE data of FOXO4^TAD^ yield differential solvent accessibilities in a residue-specific manner (Fig. 2b, c). H^α^ atoms located in regions rich in bulky residues are showing lower sPREs and H^α^ atoms located in more exposed glycine-rich regions display higher sPREs. H^β^ sPRE data was obtained for a limited number of residues and shows overall elevated sPREs due to the higher degree of exposure and a reasonable agreement of predicted and experimental data (Supporting Fig. 1). A comparison of the predicted sPRE data with a bioinformatics bulkiness prediction shows that some features are reproduced by the bioinformatics prediction (Supporting Fig. 2A). However, the experimental sPRE is better described by our approach. Strikingly, the predicted sPRE pattern reproduces the experimental sPRE pattern exceptionally well, indicating that the FOXO4^TAD^ is largely disordered and does not adopt any stable or transient tertiary structure in the regions for which sPRE data could be obtained.

In order to demonstrate that surface accessibility data can be obtained for a IDP with potential formation of transient local secondary structure we recorded sPRE data for the 94-residue transactivation domain of p53. p53 is a homo-tetrameric transcription factor composed of an N-terminal trans-activation domain, a proline-rich domain, a central DNA-binding domain followed by a tetramerization domain and the C-terminal negative regulatory domain. p53 is involved in the regulation of more than 500 target genes and thereby controls a broad range of cellular processes, including apoptosis, metabolic adaptation, DNA repair, cell cycle arrest, and senescence (Vousden and Prives 2009). The disordered N-terminal p53 transactivation domain (p53^TAD^) is a key interaction motif for regulatory protein–protein interactions (Fernandez-Fernandez and Sot 2011): it possesses two binding motifs with α-helical propensity, named p53^TAD1^ (residues 17–29) and p53^TAD2^ (residues 40–57). These two motifs act independently or in combination in order to allow p53 to bind to several proteins regulating either p53 stability or transcriptional activity (Shan et al. 2012; Jenkins et al. 2009; Rowell et al. 2012). Because of its pro-apoptotic function, p53 is recognized as tumor suppressor, and is found mutated in more than half of all human cancers affecting a wide variety of tissues (Olivier et al. 2010). Within this biological and disease context the N-terminal p53-TAD plays a key role: it mediates the interaction with folded co-factors, and comprises most of the regulatory phosphorylation sites.

**Fig. 3:**
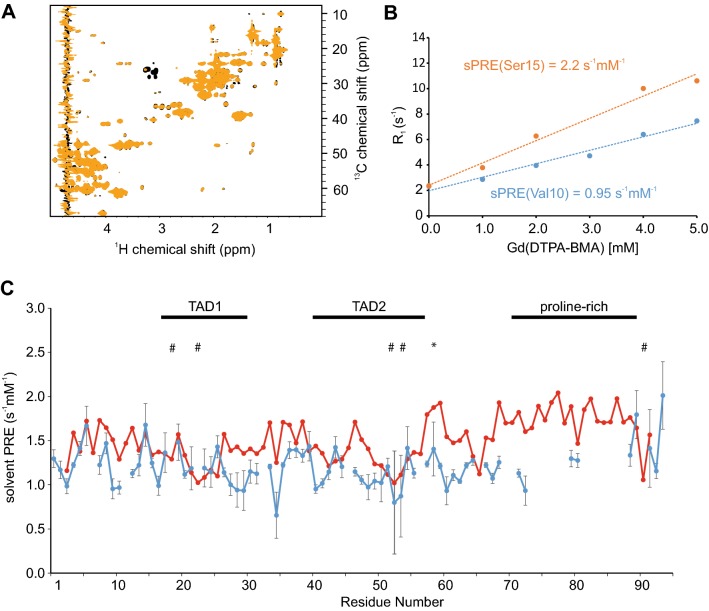
Comparison of predicted and measured solvent PRE of p53^TAD^. **a** Overlay of ^1^H, ^13^C HSQC read-out spectra, with full recovery time of a 300 µM ^13^C, ^15^N labeled p53^TAD^ in absence (black) and presence of 5 mM Gd(DTPA-BMA) (orange). **b** Gd(DTPA-BMA)-concentration-dependent R_1_ rates of two selected residues. **c** Diagram showing predicted (red) and measured (blue) solvent PRE values of each H^α^ atom of p53^TAD^. Experimental sPRE values are calculated by fitting the data with a linear regression equation. Predicted sPRE values are based on the previously described ensemble approach. Regions binding to co-factors (TAD1, TAD2) and the proline rich region are labeled. Residues with bulky side chains (Phe, Trp, Tyr) are labeled with *#*, and exposed glycine residues are labeled with * (see Supporting Fig. 2B for a bulkiness profile). Errors of the measured ^1^H-R_1_ rates were calculated using a Monte Carlo-type resampling strategy and are shown in the diagram as error bars

^1^H, ^15^N and ^1^H, ^13^C HSQC NMR spectra recorded of p53^TAD^ are of high quality and showed no detectable ^1^H, ^13^C, or ^15^N chemical shift changes between the spectra recorded in the absence or presence of Gd(DTPA-BMA) (Fig. 3a, Supporting Fig. 3A). The sPRE data of p53^TAD^ display differential solvent accessibilities in a residue-specific manner: due to different excluded volumes for the paramagnetic agent H^α^ atoms located in regions rich in bulky residues show lower sPREs and H^α^ atoms located in more exposed regions show higher sPREs (Fig. 3b, c, Supporting Fig. 2B).

sPRE data of structured proteins are often recorded for amide protons. However, chemical exchange of the amide proton with fast-relaxing water solvent protons might lead to an increase of the experimental sPRE, as has been observed for the disordered linker regions in folded proteins and in RNA (Hartlmuller et al. 2017; Gobl et al. 2017). For imino and amino protons of the UUCG tetraloop RNA and a GTP class II aptamer, for example, the increase of ^1^H-R_1_ rates is larger at small concentrations of the paramagnetic compound, and becomes linear at higher concentrations. Thus, we decided to focus here on experimental and back-calculated sPRE data of H^α^ protons. Nevertheless, ^1^H^N^-sPREs are shown for comparison in the supporting information (Supporting Fig. 4A).

Comparison of the back-calculated and experimental p53^TAD^-sPREs shows that several regions within p53^TAD^ yield lower sPREs than predicted, indicating that p53^TAD^ populates residual local structure or shows long-range tertiary interactions. In line with this, ^15^N NMR relaxation data and ^13^C secondary chemical shift data display reduced flexibility of p53^TAD^ and transient α-helical structure (Supporting Fig. 5). This is in line with previous studies which found that the p53^TAD1^ domain adopts a transiently populated α-helical structure formed by residues Phe19-Leu26 and that the p53^TAD2^ domain adopts a transiently populated turn-like structure formed by residues Met40-Met44 and Asp48-Trp53 (Lee et al. 2000). Given that p53^TAD^ has been reported to interact with several co-factors, our data indicate that sPRE data can indeed provide important insight into the residual structure of this key interaction motif (Bourgeois and Madl 2018; Raj and Attardi 2017).

In order to address the question of whether sPREs can be used to detect transient long-range interactions in disordered proteins we recorded ^1^H sPRE data for the 141-residue IDP α-synuclein using ^1^H, ^13^C and ^1^H, ^15^N, HSQC-based saturation recovery experiments at increasing concentrations of Gd(DTPA-BMA). α-Synuclein controls the assembly of presynaptic vesicles in neurons and is required for the release of the neurotransmitter dopamine (Burre et al. 2010). The aggregation of α-synuclein into intracellular amyloid inclusions coincides with the death of dopaminergic neurons, and therefore constitutes a pathologic signature of synucleinopathies such as Parkinson’s disease, dementia with Lewy bodies, and multiple system atrophy (Alafuzoff and Hartikainen 2017). Formation of transient long-range interactions has been proposed to protect α-synuclein from aggregation.

**Fig. 4:**
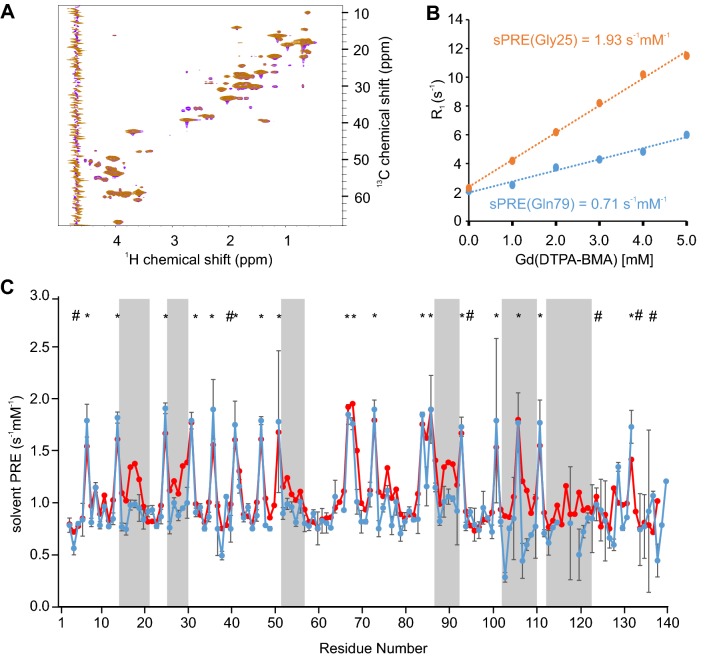
Comparison of predicted and measured solvent PRE of α-synuclein. **a** Overlay of ^1^H, ^13^C HSQC Read-out spectra, with full recovery time of 100 µM ^13^C,^15^N labeled α-synuclein in absence (violet) and presence of 5 mM Gd(DTPA-BMA) (orange). **b** Linear fit of relaxation rate ^1^H-R_1_ and Gd(DTPA-BMA) concentration of two selected residues of α-synuclein. **c** Predicted (red) and experimentally determined (blue) sPRE values from ^1^H,^13^C HSQC read-out spectra. Regions of strong variations between predicted and measured sPRE values are highlighted by grey boxes. Experimental sPRE values are calculated by fitting the data with a linear regression equation. Predicted sPRE values are based on the previously described ensemble approach. Residues with bulky side chains (Phe, Trp, Tyr) are labeled with #, and exposed glycine residues are labeled with * (see Supporting Fig. 2C for a bulkiness profile). Errors of the measured ^1^H-R_1_ rates were calculated using a Monte Carlo-type resampling strategy and are shown in the diagram as error bars

^1^H, ^15^N and ^1^H, ^13^C HSQC NMR spectra of α-synuclein are of high quality and showed no detectable ^1^H, ^13^C, or ^15^N chemical shift changes between the spectra recorded in the absence or presence of 5 mM Gd(DTPA-BMA) (Fig. 4a). The sPRE data of α-synuclein display variable solvent accessibilities in a residue-specific manner (Fig. 4b), with Hα atoms located in regions rich in bulky residues showing lower sPREs and H^α^ atoms located in more exposed regions showing higher sPREs (see also Supporting Fig. 2C for a comparison with the bioinformatics bulkiness profile and Supporting Fig. 4B for the ^1^H^N^ sPRE data). Thus, the sPRE value provides local structural information about the disordered ensemble. Strikingly, we observed decreased sPREs, and therefore lower surface accessibility, in several regions, such as between residues 15–20, 26–30, 52–57, 74–79, 87–92, 102–110, and 112–121, respectively (Fig. 4c). Comparison of these regions with recently–published ensemble modeling using extensive sets of RDC and PRE data (Salmon et al. 2010) shows that the previously–observed transient intra-molecular long-range contacts involving mainly the regions 1–40, 70–90, and 120–140 within α-synuclein are reproduced by the sPRE data. Thus, sPRE data are highly sensitive to low populations of residual structure in disordered proteins.

## Conclusions

In order to understand the conformational behavior of IDPs and their biological interaction networks, the detection of residual structure and long-range interactions is required. The large number of degrees of conformational freedom of IDPs require extensive sets of experimental data. Here, we provide a straightforward approach for the detection of residual structure and long-range interactions in IDPs and show that sPRE data contribute important and easily-accessible restraints for the investigation of IDPs. Our data indicate that for the general case of an unfolded chain with a local flexibility described by the overwhelming majority of available combinations, sPREs can be accurately predicted through our approach. It can be envisaged that a database of all potential combinations of the 20 amino acids within the central 5-mer peptide can be generated in the future. Nevertheless, generation of sPRE datasets for the entire 3.2 million possible combinations is beyond the current computing capabilities.

Our approach promises to be a straightforward screening tool to exclude potential specific interactions of the soluble paramagnetic agent with IDPs and to guide positioning of covalent paramagnetic spin labels which are often used to detect long-range interactions within IDPs (Gobl et al. 2014; Clore and Iwahara 2009; Otting 2010; Jensen et al. 2014). Paramagnetic spin labels are preferable placed close to, but not within regions involved in transient interactions in order to avoid potential interference of the spin label with weak and dynamic interactions.

In summary, we used three highly disease-relevant biological model systems for determining the solvent accessibility information provided by sPREs. This information can be easily determined experimentally and agrees well with the sPREs predicted for non-exchangeable protons using our grid-based approach. Our method proves to be highly sensitive to low populations of residual structure and long-range contacts in disordered proteins. This approach can be easily combined with ensemble-based calculations such as implemented in flexible-meccano/ASTEROIDS (Mukrasch et al. 2007; Nodet et al. 2009), Xplor-NIH (Kooshapur et al. 2018), or other programs (Estana et al. 2019) to interpret residual structure of IDPs quantitatively and in combination with complementary restraints obtained from RDCs and PREs. In particular for IDP ensemble calculations relying on sPRE data it is essential to exclude specific interactions of the paramagnetic agent with the IDP of interest which would lead to an enhanced experimental sPRE compared to the predicted sPRE.

## Electronic supplementary material

Below is the link to the electronic supplementary material.

Supporting material 1 (PDF 908 kb)
