# Supplementary material for: NMR characterization of solvent accessibility and transient structure in intrinsically disordered proteins
Source: J Biomol NMR. 2019 Jul 11;73(6):305–17. doi: 10.1007/s10858-019-00248-2 (PMC6692294; doi:10.1007/s10858-019-00248-2)
Supplement: Supplementary file 1 — Supporting material 1 (PDF 908 kb) [file 10858_2019_248_MOESM1_ESM.pdf]

NMR Characterization of Solvent Accessibility and Transient Structure in Intrinsically Disordered Proteins

Christoph Hartlmüller[1,#], Emil Spreitzer[2,#], Christoph Göbl[3], Fabio Falsone[4], Tobias Madl[1,5,*]

Supporting Information

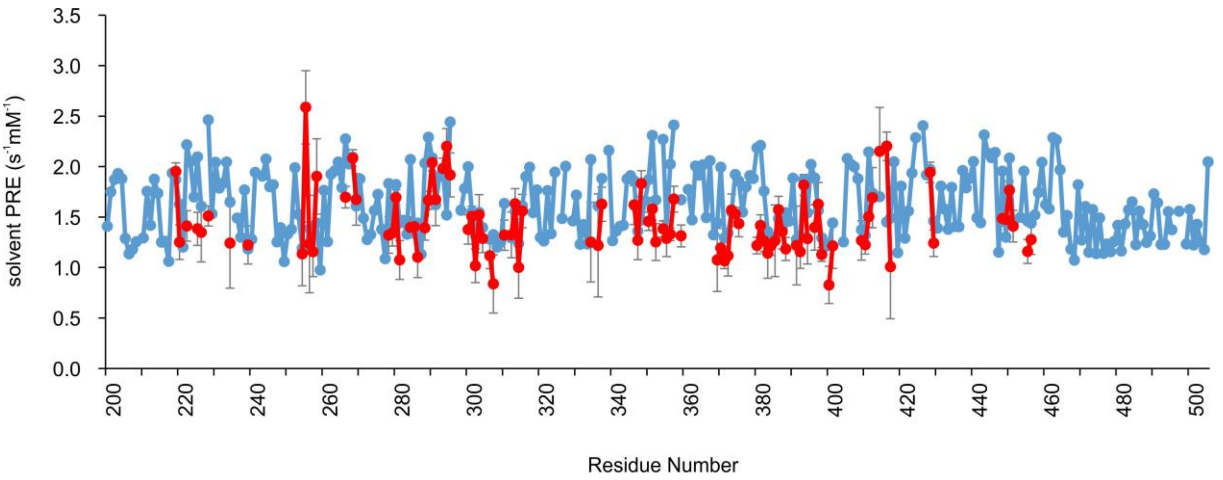

Supporting Figure 1: Experimentally-determined (red) and predicted (blue) solvent PRE values using CBCA(CO)NH as readout spectrum, of assigned H^{β} peaks of $FOXO4^{TAD}$. Experimental sPRE values are calculated by fitting the data with a linear regression equation. Errors of the measured ^{1}H-R_1 rates were calculated using a Monte Carlo-type resampling strategy and are shown in the diagram as error bars.

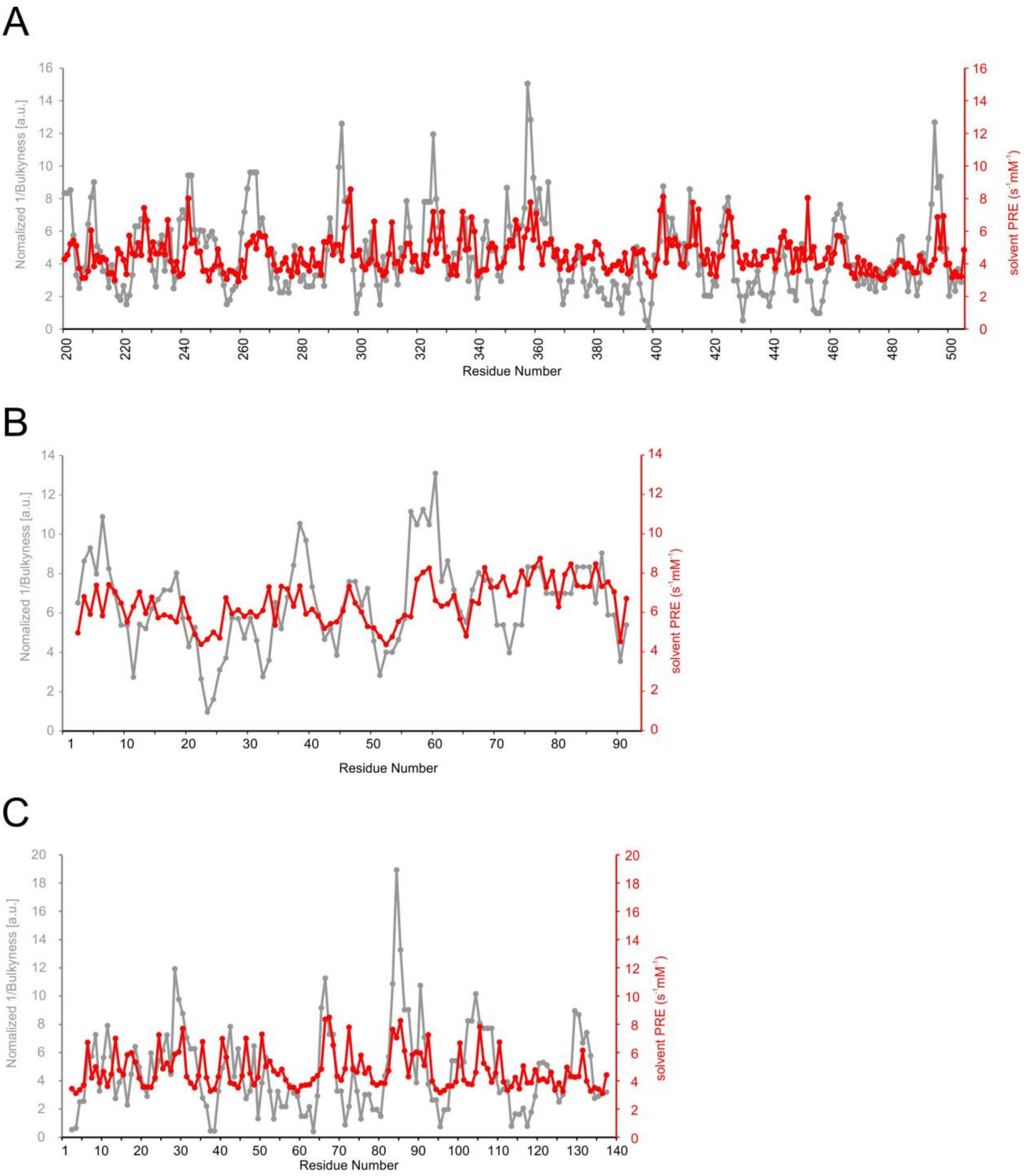

Supporting Figure 2: Comparison of experimental sPRE values of Hα protons (red) and bulkiness (grey) of FOXO4 **(A)**, p53^{TAD} **(B)** and α-synuclein **(C)**. Bulkyness was calculated using the ProtScale web server (https://web.expasy.org/protscale/, 01.04.2019) using a window size of five residues and standard parameters. To compare bulkiness and sPRE the bulkiness was inverted, a linear fit calculated and the bulkiness shifted and scaled to the predicted sPRE data.

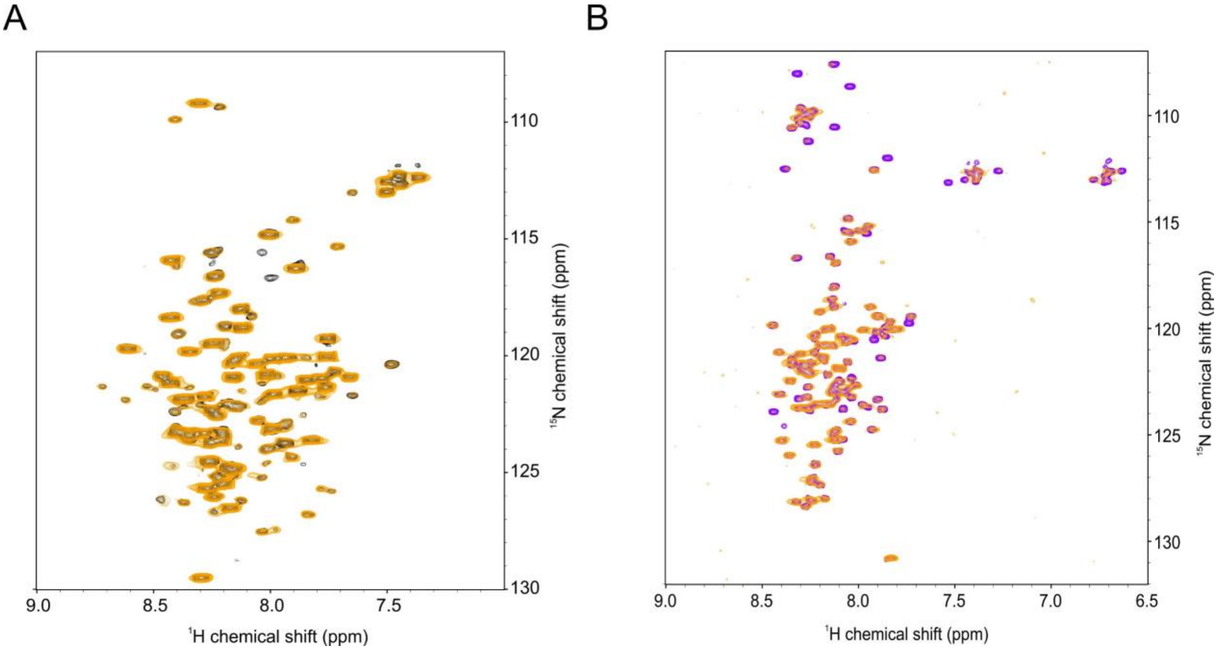

Supporting Figure 3: (A) Overlay of $^{1}H,^{15}N$ HSQC spectra, with full recovery time of a 300 µM $^{13}C,^{15}N$ labeled $p53^{TAD}$ sample in the absence (black) and presence of 3.25 mM Gd(DTPA-BMA) (orange). **(B)** Overlay of ^{1}H, ^{15}N HSQC spectra, with full recovery time of 100 µM $^{13}C,^{15}N$ labeled α-synuclein in absence (violet) and presence of 5 mM Gd(DTPA-BMA) (orange).

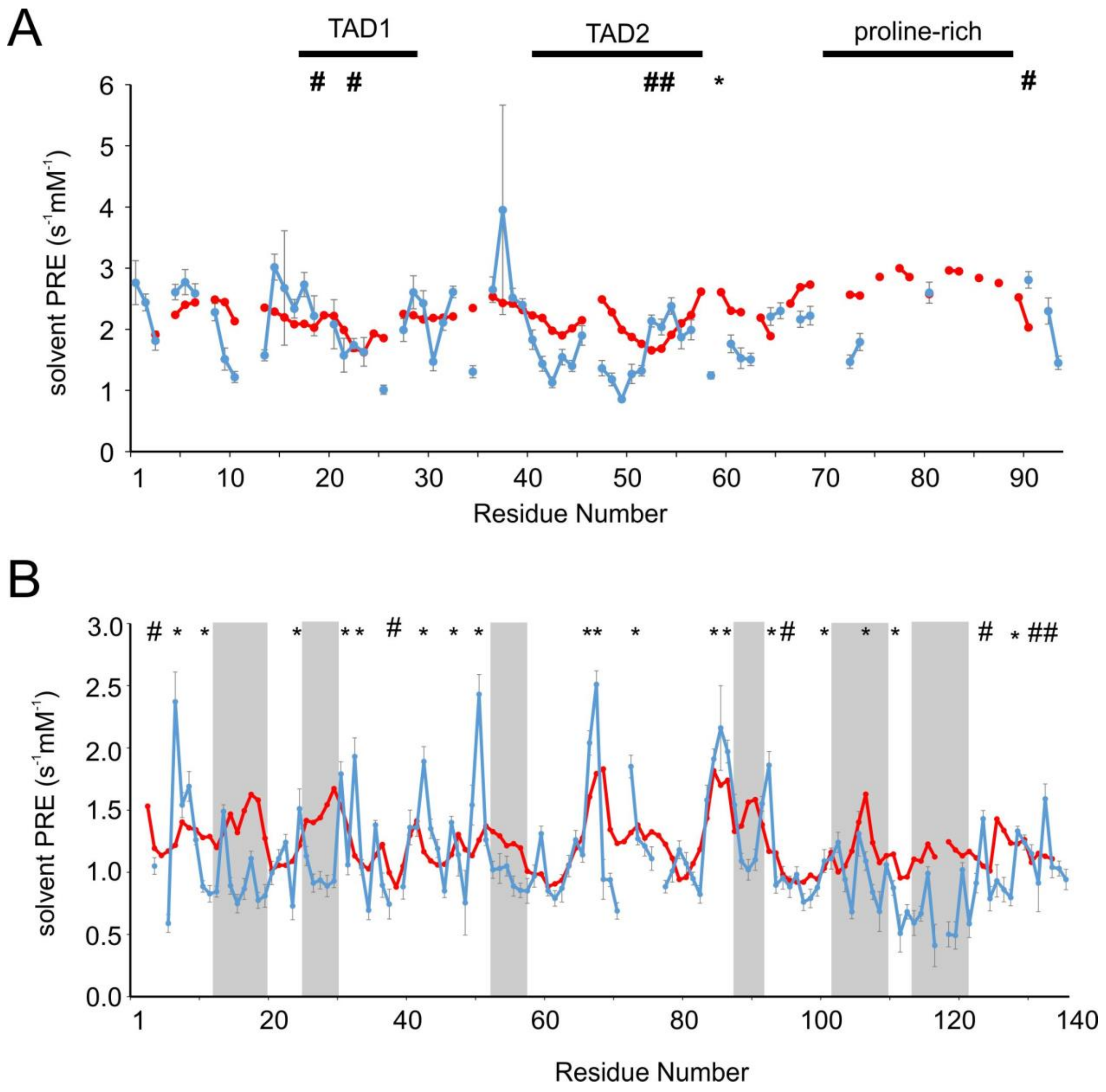

Supporting Figure 4: Comparison of predicted and measured $^1H^N$ solvent PREs of $p53^{TAD}$ (A) and α–synuclein (B). Predicted (red) and experimentally determined (blue) sPRE values from $^1H,^{15}N$ HSQC read-out spectra are shown. Regions binding to co-factors (TAD1, TAD2) and the proline rich region are labeled ($p53^{TAD}$). Regions of strong variations between predicted and measured sPRE values are highlighted by grey boxes and reproduce the shielding observed for the $^1H^\alpha$ sPRE data (α-synuclein). Experimental sPRE values are calculated by fitting the data with a linear regression equation. Predicted sPRE values are based on the previously described ensemble approach. Residues with bulky side chains (Phe, Trp, Tyr) are labeled with #, and exposed glycine residues are labeled with * (see Supporting Figure 2 for bulkiness profiles). Errors of the measured ^1H-R_1 rates were calculated using a Monte Carlo-type resampling strategy and are shown in the diagram as error bars.

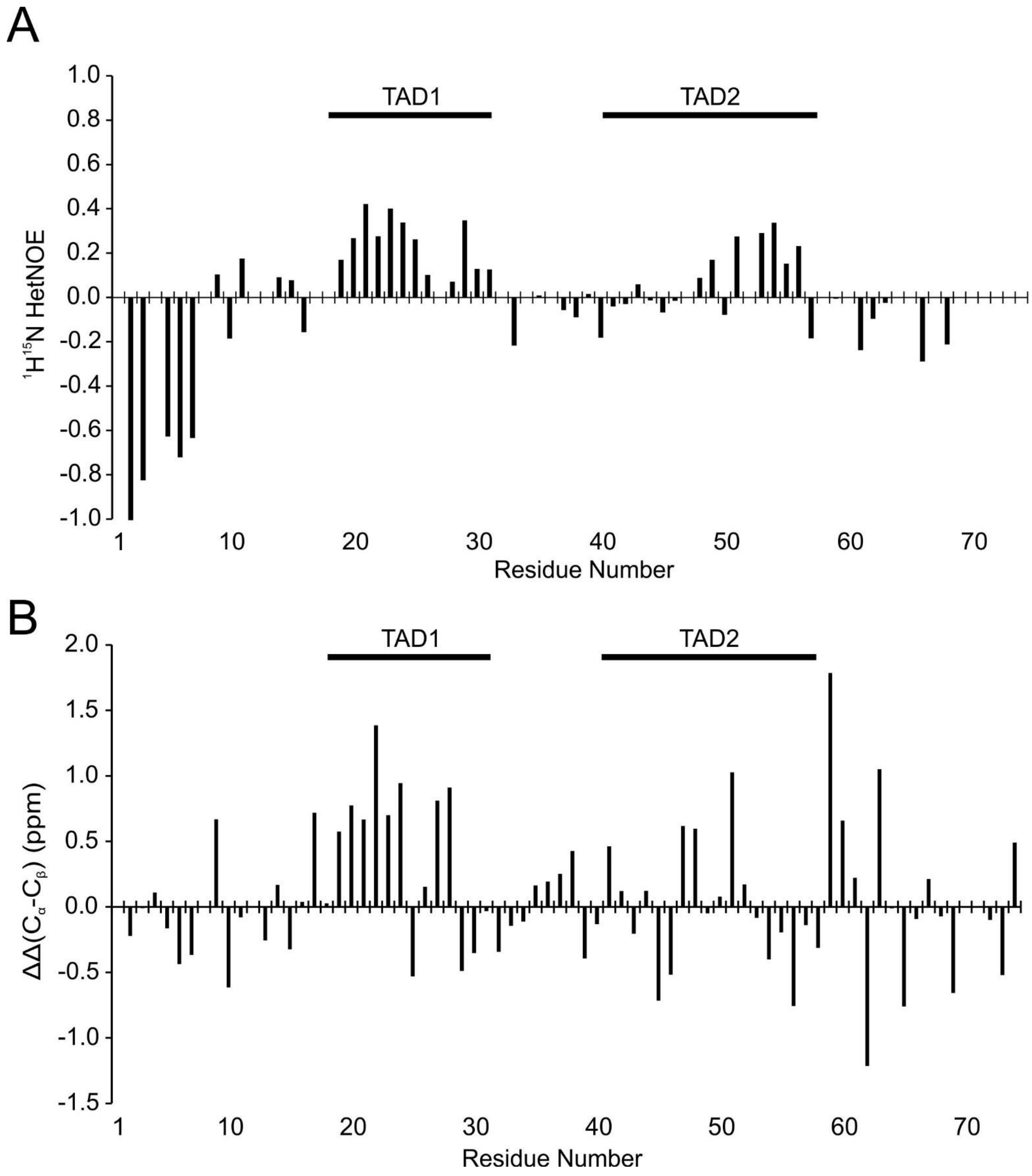

Supporting Figure 5: p53^{TAD} {^{1}H}-^{15}N heteronuclear NOE (A) and secondary chemical shifts (B). {^{1}H}-^{15}N heteronuclear NOEs were recorded on an Avance Neo 600 MHz spectrometer using the same conditions as described in materials and methods. Secondary chemical shifts were calculated from $^{13}C^{\alpha}$ and $^{13}C^{\beta}$ chemical shifts obtained from backbone resonance assignment.
